# Effect of an optimized X-ray blanket design on operator radiation dose in cardiac catheterization based on real-world angiography

**DOI:** 10.1371/journal.pone.0277436

**Published:** 2022-11-10

**Authors:** Cedric Davidsen, Kirsten Bolstad, Kristian Ytre-Hauge, Andreas Tefre Samnøy, Kjell Vikenes, Vegard Tuseth

**Affiliations:** 1 Department of Heart Disease, Haukeland University Hospital, Bergen, Norway; 2 Department of Cardiology, CHU Sart Tilman, Liège University Hospital, Liège, Belgium; 3 Department of Oncology and Medical Physics, Haukeland University Hospital, Bergen, Norway; 4 Department of Physics and Technology, University of Bergen, Bergen, Norway; 5 Department of Clinical Medicine, University of Bergen, Bergen, Norway; Baylor Scott and White, Texas A&M College of Medicine, UNITED STATES

## Abstract

**Background:**

There is increasing concern and focus in the interventional cardiology community on potential long term health issues related to radiation exposure and heavy wearable protection. Optimized shielding measures may reduce operator dose to levels where lighter radioprotective garments can safely be used, or even omitted. X-ray blankets (XRB) are commercially available but suffer from small size and lack of stability. A larger XRB may reduce operator dose but could hamper vascular access and visualization. The aim of this study is to assess shielding effect of an optimized XRB during cardiac catheterization and estimate the potential reduction in annual operator dose based on DICOM Radiation Dose Structured Report (RDSR) data reflecting everyday clinical practice.

**Methods:**

Data accumulated from 7681 procedures over three years in our RDSR repository was used to identify projection angles and radiation doses during cardiac catheterization. Using an anthropomorphic phantom and a scatter radiation detector, radiation dose to the operator (mSv) and patient (dose area product—DAP) was measured for each angiographic projection for three different shielding setups. Relative operator dose (mSv/DAP) was calculated and multiplied by DAP per projection to estimate effect on operator dose.

**Results:**

Adding an optimized XRB to a standard shielding setup comprising a table- and ceiling-mounted shield resulted in a 94.9% reduction in estimated operator dose. The largest shielding effect was observed in left and cranial projections where the ceiling-mounted shield offered less protection.

**Conclusions:**

An optimized XRB is a simple shielding measure that has the potential to reduce operator dose.

## Introduction

During coronary angiography and percutaneous coronary intervention (PCI), the patient and operator are both exposed to ionizing radiation. The operator is not exposed to the primary beam, but to radiation that occurs when a small amount of the photons that reach the patient are scattered towards the operator. Although the operator dose is only a fraction of the patient dose during a given procedure, the operator may perform hundreds of procedures each year [1], and there are growing concerns about the potential negative health effects of repeated exposure over many years of professional life as an interventional cardiologist. In addition, staff exposed to >1 mSv per year is required to wear radioprotective clothing during procedures. They do, however, not protect the operators’ head or extremities, are heavy, and may lead to orthopedic strain injuries over time [2]. Improving the shielding around the source of scatter is thus a particularly attractive option as it would also protect areas not covered by radioprotective garments and may reduce operator dose to levels where lighter protection can be worn.

An ideal setup to fully protect the operator from radiation exposure would be a continuous X-ray shielding wall between the patient and operator. Some comprehensive solutions have been proposed [3], but with limited uptake amongst interventional cardiologists. In everyday practice the most common setup is a combination of table- and ceiling-mounted lead shields. This setup tends to leave a gap at patient level and recently there has been an increased focus on placing a X-ray blanket (XRB) on the patient to improve shielding continuity (S1 Fig). Clinical trials have found highly variable effect with reductions in operator dose ranging from 20 to 76% [4–10], but size, design, shielding properties and positioning of the blanket were not standardized. Available XRBs are of limited size which may limit the protective effect. A larger blanket may improve shielding but could hamper access and visualization as well as handling. Based on real time personal dosimetry, phantom pilot measurements and clinical pilots, we designed a customized lead blanket that would maximize coverage area, while retaining flexibility of vascular access and ease of use for the operator.

For assessing clinical efficacy of radiation shielding measures used during cardiac catherization, it is important to incorporate the multiple projections needed to properly visualize the coronary arteries as this strongly influences operator dose [11]. Yet, little data is available on projections used in everyday practice nor how they influence the protective effect of an XRB. In this study, we use data from a large number of real-world procedures to determine which C-arm angulations are used and in which proportion. This information is then used to test the XRB in a controlled standardized setup mirroring everyday clinical practice to estimate annual operator dose reduction potential.

## Material and methods

### The XRB

Preliminary pilots indicated that XRB size and positioning were critical for operator protection. The optimal position of the XRB was found to be as cranially as possible without impeding on the imaging area (S2 Fig). Also from the initial pilots, we concluded a 60 cm x 60 cm format would represent a good balance between patient cover, being light enough to handle and small enough to fit inside a sterile plastic cover if needed. A prototype was created using a CE-marked XRB with lead equivalency of 0.5mm (Scanflex Medical AB). The prototype was informally tested during clinical procedures with encouraging real-time dosimetry readings.

### Real-world cath lab dose and projections

Radiation data at our institution are stored in OpenREM which is an opensource PostgreSQL database that stores DICOM Radiation Dose Structured Report (RDSR) data for each procedure. These RDSR data contain key details from each exposure such as C-arm angulation, dose area product (DAP) and imaging geometry. Data were extracted from 7681 procedures performed at three cath labs in our institution between February 2017 and March 2020. As the data were fully anonymized and consisting of retrospective procedural data from a large number of procedures with no identifiable personal health data, the regional ethics board waived the requirement for informed consent. Radial approach was used in >80% of cases. PCI was performed in approximately 40% of cases and the data also include weekly CTO (chronic total occlusion) sessions. Median fluoro time per procedure was 470 seconds (IQR 218–943 seconds) and median cine duration 39 seconds (IQR 28–58 seconds). Mean and median DAP per procedure was 36101 mGycm^2^ and 24129 mGycm^2^ (IQR 12818–45209 mGycm^2^). Exposures in different projection angles were grouped into AP, CRAN, CAUD, LAO, LAO-CRAN, LAO-CAUD, RAO, RAO-CRAN and RAO-CAUD and LAO90. Angiographic projection grouping categories were defined so that unidirectional projections such as LAO or RAO also included +/-10° in the cranio-caudal direction, CAUD and CRAN +/-10° in the left-right direction and AP +/- 10° in any direction. For each projection, the total accumulated DAP and fraction of total accumulated DAP was calculated. The underlying data set of DAP and C-arm angulation for each exposure is available as S1 File.

### Cath lab measurement setup

**Fig 1:**
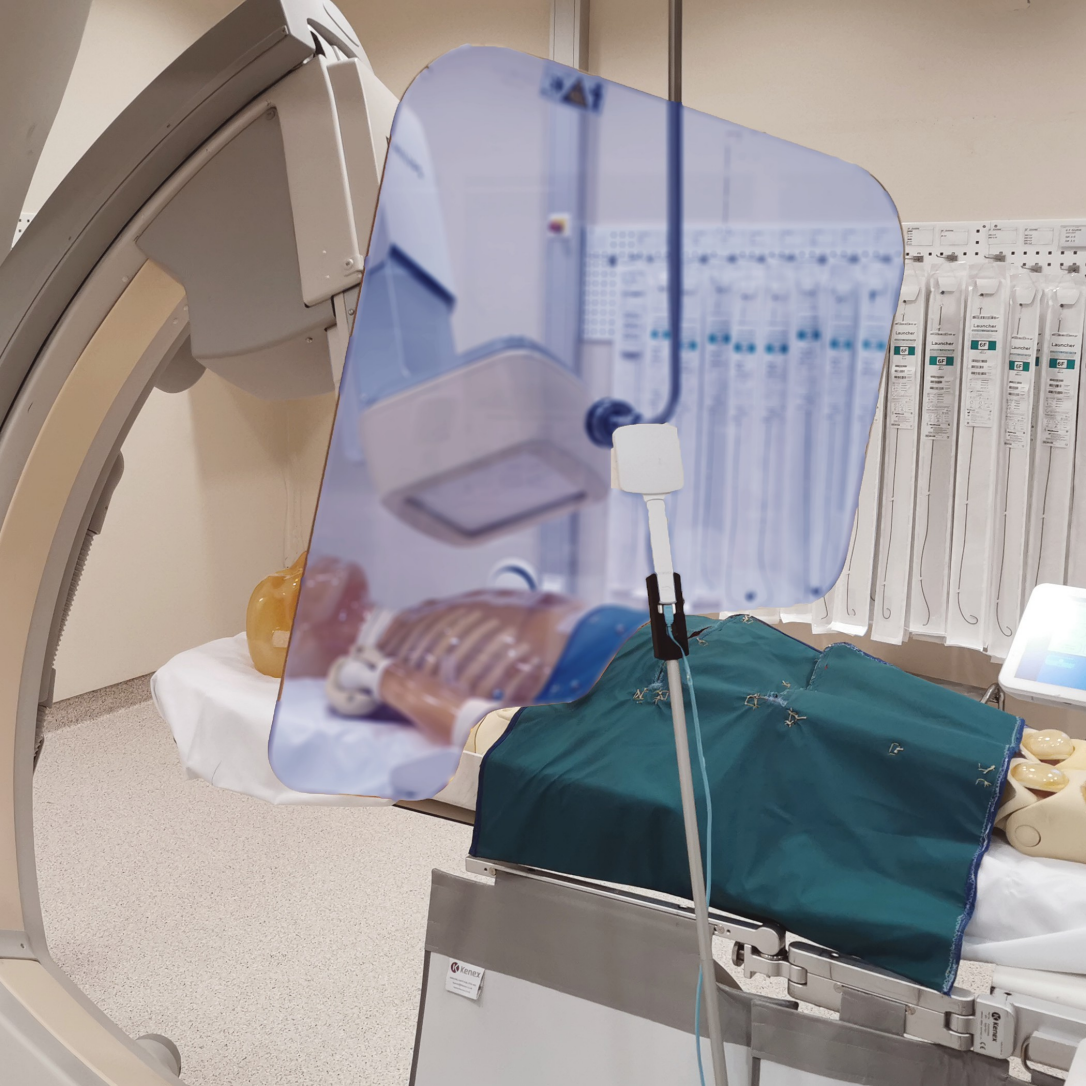
Illustration of measurement setup. A 60x60cm X-ray blanket (flexible features not shown) is positioned just caudally to the image detector, and the X2 Survey sensor in the center of the photo is placed 140cm above the floor, 40cm caudally and laterally to the center of the primary beam. This corresponds to the position of the operator’s left shoulder during cardiac catheterization using a right radial approach.

Measurements were done in a cath lab equipped with a Philips Allura Xper FD10C C-arm from 2009 where the X-ray source is located 33.5 cm above floor level. Table height was set to "0 cm" where the lower edge of the table is 88.5 cm above the floor, or 55 cm above the X-ray source. Source-to-image distance (SID) of 100 cm was used, 20x20 cm^2^ field of view and 15 frames per second cine protocol. A high framerate cine protocol was chosen as it produces sufficient scatter radiation for reliable measures. To simulate the patient, a Kyoto Kagaku Whole Body Phantom PBU-50 corresponding to a person measuring 165 cm and 50kg was used. Protective elements included a ceiling-mounted Mavig OTS54011 lead acrylic X-ray shield and a Kenex 312/DS-039/5 table-mounted lower body X-ray shield, both providing 0.5mm lead equivalent protection. Scatter radiation was measured with a Raysafe X2 Survey Sensor placed 140 cm above floor level, and 40 cm caudally and laterally to the center of the primary beam (Fig 1). This position corresponds to a dosimeter worn on the left shoulder of an operator measuring 180 cm standing close to the patient as is the case in radial procedures. The X2 has a directional sensor with backscatter protection on the back. During measurements, it was directed towards the patient. For each measurement, operator dose was measured with the X2 sensor, whereas patient dose, DAP, was collected from the C-arm.

### Projections and XRB shielding effect

Three setups were compared: 1) A “no shielding” setup with only the table-mounted shield, 2) a “standard” shielding setup with ceiling-mounted shield in addition to the table-mounted shield, and 3) an optimized shielding setup named “XRB” where an adequately sized and positioned XRB was added to the standard setup (Fig 1). When used, the ceiling-mounted shield was positioned 10 cm caudally to the imaging detector and rotated 30° around its vertical axis so the lateral edge would be more cranial than the medial edge. In the horizontal plane the ceiling-mounted shield was positioned 5 cm above the patient surface which represents a realistic real-world positioning since in clinical practice it is difficult to position the shield in direct contact with the patient. The XRB was positioned directly caudally to the imaging detector. For each projection, the mean angle in the left-right and cranio-caudal directions obtained from our RDSR depository was used, and measurements were repeated five times.

### Calculation of shielding effect

Operator dose is measured in millisievert (mSv) whereas patient dose is quantified in Dose Area Product (DAP in mGycm^2^). The relationship between these is described by the relative operator dose which is the ratio between operator dose in mSv and patient DAP measured in mGycm^2^. It is a validated parameter for assessing effect of radiation protection devices in invasive cardiology [12]. As it normalizes received operator dose to given patient DAP, it allows for direct comparisons regardless of irradiation duration or imaging protocol. It is important to acknowledge that when tilting the C-arm, the amount of patient tissue between the X-ray source and detector increases, and the X-ray system will automatically adapt tube current and voltage to maintain image quality. Thus, to make correct comparisons between angiographic projections, it is necessary to correct for this variation by dividing received operator dose by given patient DAP.

### Annual operator dose reduction with an XRB

To assess potential effect on real life annual operator dose based on mSv/DAP ratio measurements for the different shielding setups, clinical DAP readings were extracted from OpenREM. Clinical DAP was distributed to projections according to the proportion in which they were used and DAP per year per operator was calculated. Yearly DAP per operator was then multiplied with the mSv/DAP ratios for all projections in the different shielding setups for estimating annual operator dose.

### Data analysis

Data analysis was done in RStudio: integrated development for R version 1.1.456 (RStudio, Inc., Boston, MA). Plots were created with the ggplot2 version 3.3.3 package. The corresponding author had full access to all the data in the study and takes responsibility for its integrity and the data analysis.

## Results

### Real-world cath lab dose and projections

**Fig 2:**
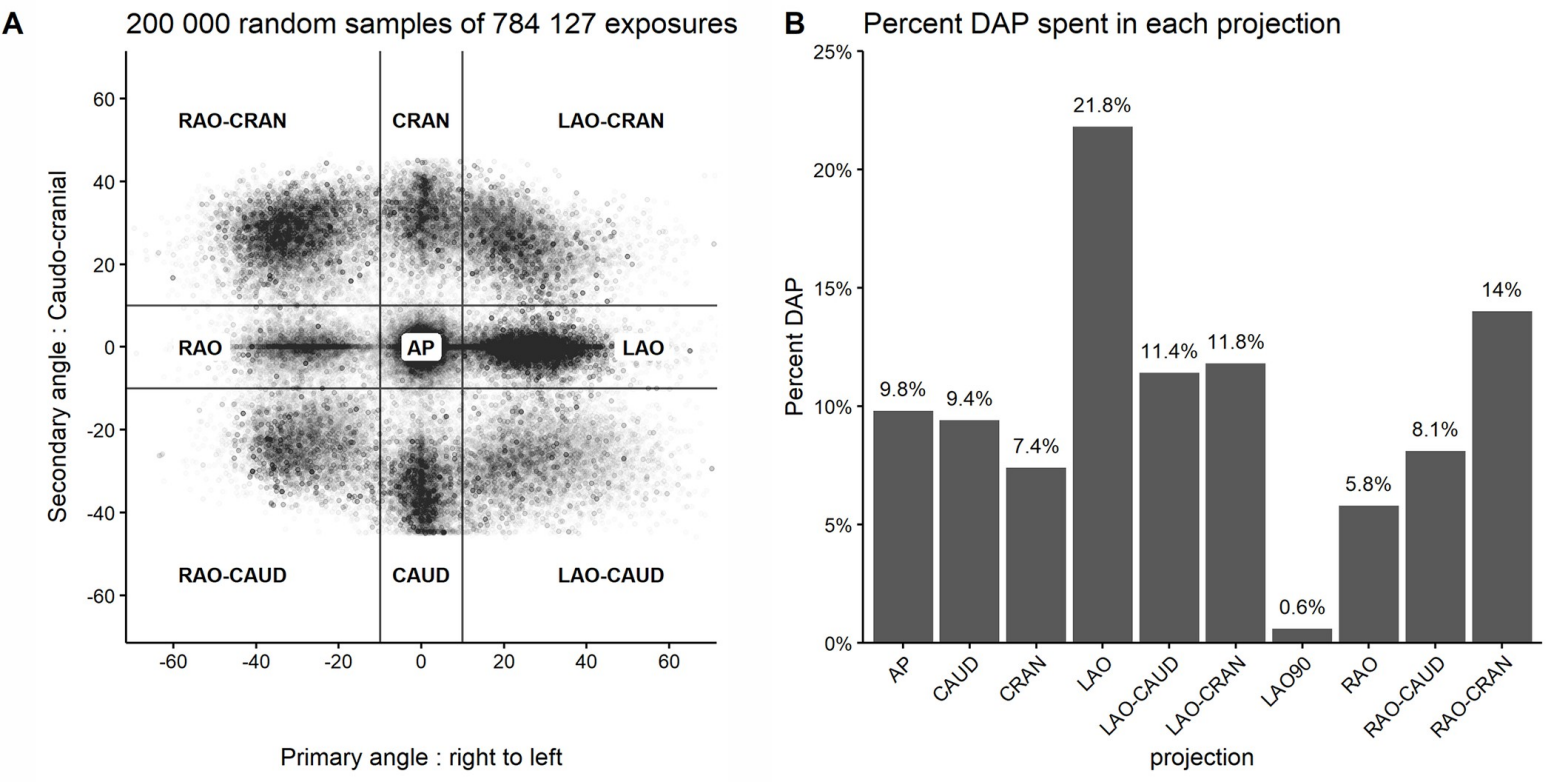
C-arm angulation and percentage DAP in each projection. Panel A: Scatterplot showing the precise C-arm angulation of 200 000 random samples out of 784 154 exposures. Only a sample was plotted to avoid overplotting and improve visualization. Although a large variation in C-arm angulation is present, it is easy to visualize the natural grouping categories. Panel B: Percentage DAP recorded in each projection. LAO (21.4%) and RAO-CRAN (14%) are where most patient doses are given.

Fig 2A is a scatterplot of a random sample of 200 000 exposures from our RDSR data that illustrates the variation in C-arm projection angle used, as well as visual fit according to grouping categories. The percent DAP spent in each projection is presented in Fig 1B. LAO (21.8% of all DAP) was most commonly used, followed by RAO-CRAN (14%) and LAO-CRAN (11.8%). The least used were LAO90 (0.8%), RAO (5.8%) and CRAN (7.4%). For each projection group we summarized the number of exposures, percent DAP and mean angle in the cranio-caudal and left-to right direction (S1 Table).

### Projections and XRB shielding effect

**Fig 3:**
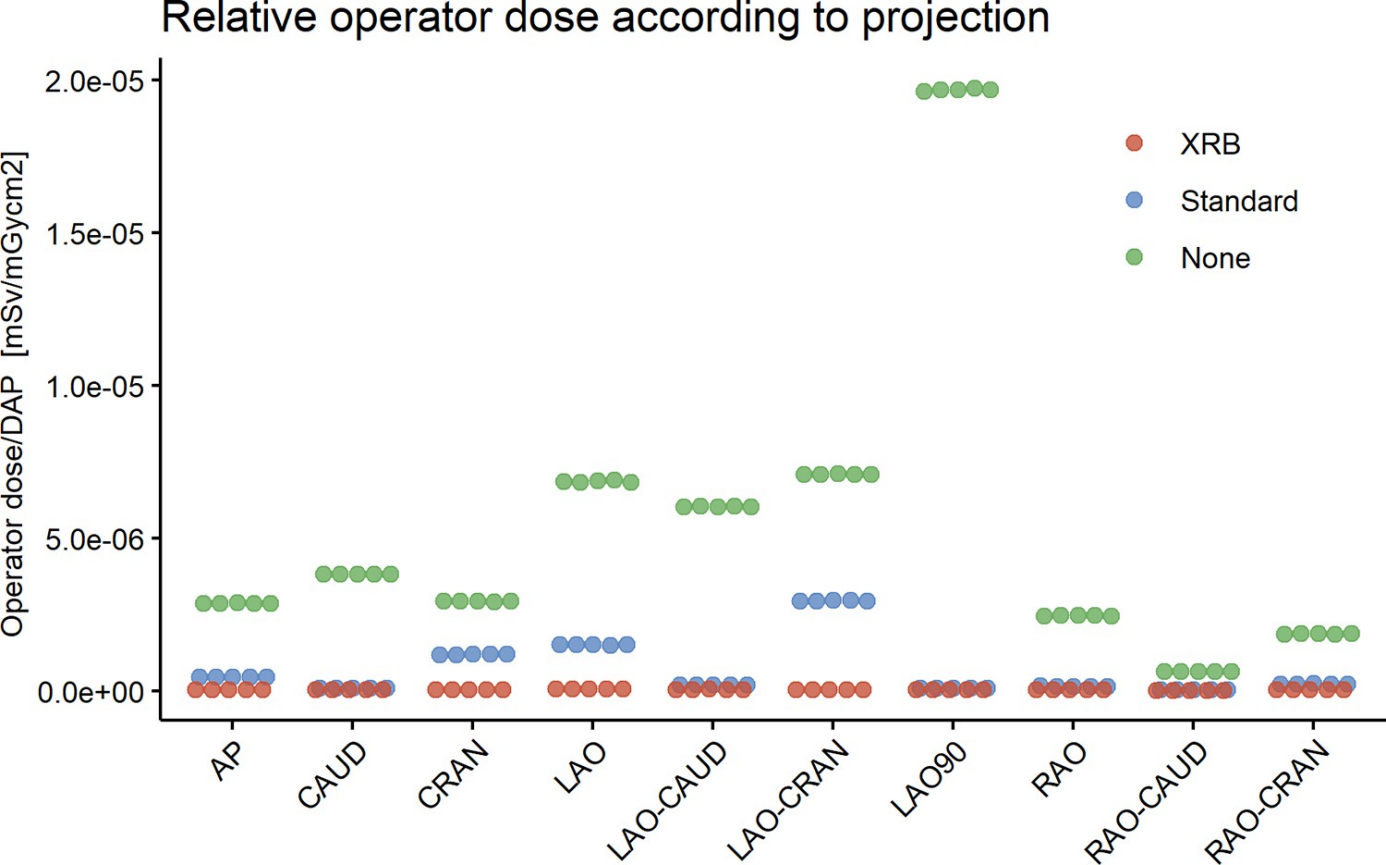
Relative operator dose according to angiographic projection and shielding setup. Each measurement was repeated five times and all measured values are individually plotted. The plot shows that standard shielding is least effective in left and cranial projections (CRAN, LAO, LAO-CRAN), whereas with the XRB the relative operator dose is consistently low. Thus, the XRB is more effective in the projections where the standard shielding has least effect.

The relative operator dose of each measurement according to shielding setup and projection group is plotted in Fig 3 and the corresponding numeric values as well as percent reduction between shielding setups are available in Table 1. As illustrated in Fig 3, the values recorded per setup and projection were very consistent with only minor variation between measurements. In the no shielding setup, LAO90 (normalized to 1) resulted in the highest relative operator dose, followed by LAO-CRAN (0.36) and LAO (0.35), whereas RAO-CAUD (0.03), RAO-CRAN (0.10) and RAO (0.12) yielded the lowest relative operator dose. Adding a standard shielding setup resulted in a reduction in relative operator dose across all projections, but the reduction was highly variable. It was least effective in LAO-CRAN (-58.4%), CRAN (-59.2%) and LAO (-78%), whereas it was more effective in the right and caudal projections. Thus, in this setup, the highest relative operator dose was seen in three projections accounting for 41% of all DAP (LAO-CRAN (normalized to 1), LAO (0.51) and CRAN (0.40)).

**Table 1:** Relative operator dose according to angiographic projection and shielding setup.

	Relative operator dose (mSv/mGycm^2^)			Reduction in relative operator dose		
Projection	None	Standard	XRB	Standard vs None	XRB vs Standard	XRB vs None
AP	2.9E-06	4.5E-07	3.9E-08	-84.4%	-91.2%	-98.6%
CAUD	3.8E-06	8.7E-08	3.9E-08	-97.7%	-54.6%	-99%
CRAN	2.9E-06	1.2E-06	4.0E-08	-59.2%	-96.7%	-98.6%
LAO	6.9E-06	1.5E-06	6.4E-08	-78%	-95.8%	-99.1%
LAO-CAUD	6.0E-06	1.9E-07	5.5E-08	-96.9%	-70.7%	-99.1%
LAO-CRAN	7.1E-06	3.0E-06	5.2E-08	-58.4%	-98.2%	-99.3%
LAO90	2.0E-05	8.7E-08	3.3E-08	-99.6%	-62.4%	-99.8%
RAO	2.5E-06	1.5E-07	3.3E-08	-93.7%	-78.9%	-98.7%
RAO-CAUD	6.3E-07	4.4E-08	1.7E-08	-93%	-62.4%	-97.4%
RAO-CRAN	1.9E-06	2.4E-07	3.4E-08	-87.4%	-85.5%	-98.2%

XRB = X-ray blanket. Standard = standard shielding setup.

Adding an XRB resulted in an additional reduction in relative operator dose. As seen in Fig 3, the reductions followed a complementary pattern where the XRB was the most effective in the projections where the ceiling-mounted shield was less effective. In LAO-CRAN, reduction in relative operator dose was -98.2%, in CRAN -96.7% and in LAO -95.8% whereas it had least additional shielding effect in CAUD (-54.6%) and RAO-CAUD (-62.4%). The resulting effect was that with an XRB, the relative operator dose was consistently low, with small variations between projections.

### Annual operator dose reduction with an XRB

**Fig 4:**
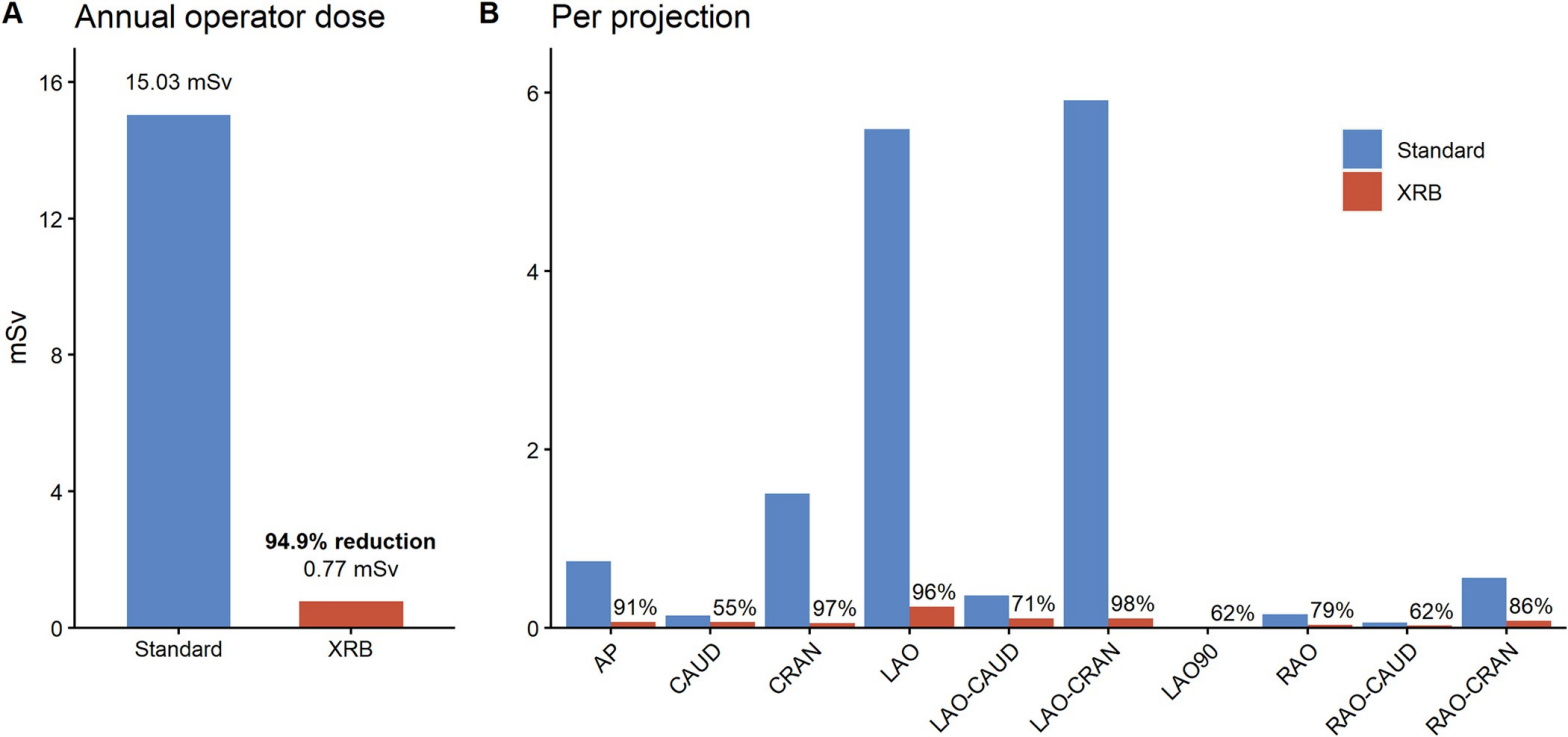
Annual operator dose estimates according to shielding setup. Calculations are based on a case load of 500 procedures / year and mean DAP per procedure 36 102 mGycm^2^. A: Adding an X-ray blanket (XRB) to standard shielding resulted in a 94.9% reduction in annual operator dose. B: Contribution of each projection to annual operator dose. The percentage above the red columns represent percent reduction with an XRB compared to standard shielding. In the standard setup, CRAN, LAO and LAO-CRAN are responsible for the majority (86%) of operator dose. These are the projections where the ceiling-mounted shield is least effective and where adding an XRB leads to the largest incremental reduction in operator dose.

To estimate operator dose, it is necessary to combine given patient dose (DAP) with the relative operator dose (operator dose/DAP) in each projection according to shielding setup. In our hospital, a full-time consultant will on average perform approximately 500 procedures per year. Annual DAP per operator was estimated by multiplying case load by mean DAP per procedure from our RDSR repository. DAP was distributed to each projection according to the percentage in which it was used (Fig 2) then multiplied with measured operator dose/DAP (Fig 3) according to shielding setup (Fig 4, S2 Table). For the XRB shielding setup, calculated annual operator dose would be 0.77 mSv. If standard shielding was used, annual operator dose would be 15.03 mSv, and with no shielding 75.53 mSv. Thus, adding an optimally placed XRB to a standard shielding setup resulted in an estimated 94.9% reduction in yearly operator dose compared to standard shielding. Fig 4B examines the relative contribution of each projection to the annual operator dose. With standard shielding, CRAN, LAO-CRAN, and LAO are responsible for 86% of annual operator dose, as these projections are both frequently used (41% of all DAP) and where standard shielding is least effective.

## Discussion

Our data show that adding an XRB to a standard shielding setup has the potential to substantially reduce operator radiation dose during cardiac catheterization. However, shielding effect is highly variable in the different angiographic projections.

### Real-world cath lab dose and projections

Coronary angiography and PCI are dynamic procedures which require multiple angiographic projections to properly examine the three-dimensional anatomy of the coronary arteries with a two-dimensional imaging system. Each patient is unique, and depending on which artery needs treatment, the optimal C-arm position will be different. In everyday practice, the C-arm is positioned to the desired angle by the operator or assisting radiographer. This has the advantage that if visualization is suboptimal, the operator can easily adapt the position of the C-arm, but also means there will be a large variation in which C-arm angulations are used. Although there are publications that have tried to establish a set of angiographic projections that minimize patient and operator dose [13], little is known about what is done in routine clinical practice. This is important to address as C-arm angle influences the patient and operator dose by several folds. Through our RDSR data repository we were able to analyze a large number of procedures and establish a reference for angiographic projection angles and in which proportion they are used during a procedure. As expected, and as illustrated in Fig 2A, there is a large variation in C-arm angulations. To our knowledge, it is the first time this type of data has been published. Not surprisingly, LAO is the most commonly used projection as it is used for positioning the diagnostic catheters and gives a good visualization of the three segments of the right coronary artery. The CRAN and LAO-CRAN are also extensively used to visualize left anterior descending artery and are particularly useful for bifurcation lesions affecting diagonal branches. Our data suggest that these three common projections represent a substantial proportion (41%) of the given radiation in clinical practice. Such findings are of particular relevance when assessing the effect of different operator shielding measures as indicated in this study.

### Shielding element size, positioning, and operator dose

Our measurements indicate striking effect of adding an XRB to existing shielding but warrant sufficient size and optimal positioning. This implies adequate coverage of the relevant field of scatter as well as placing the blanket as cranially as possible without impeding on the imaging detector. In cardiac catherization, the interface between the patient and ceiling-mounted shield is particularly vulnerable when table height and position are shifted during procedures. In this regard it is important to remember that interventional cardiologists often work in a stressful setting where a meticulous repositioning of shielding elements cannot be expected. In our experience, if the XRB is well-placed at the start of the procedure it will not compromise the images in the standard views, and no repositioning was needed during measurements on the anthropomorphic phantom. Interestingly, our initial investigations suggest that a well-positioned XRB will counteract the effect of a gap between the patient and the ceiling-mounted shield. On the contrary, if the XRB is placed too caudally, the shielding effect is quickly lost.

### Projections and XRB shielding effect

Our data show that with no shielding, left and cranial angulations of the C-arm expose the operator to proportionally larger amounts of scatter radiation. This was expected, since when the detector is tilted cranially or to the left, the under-the-table X-ray source comes closer to the operator and thus increases scatter radiation to the operator. This has previously been described in the literature [11], but to our knowledge how this influences the shielding effect of a ceiling-mounted shield or an XRB has not been evaluated. What our measurements add to current knowledge is that the ceiling-mounted shield have a limited shielding effect in left and cranial projections. The addition of a well-positioned adequately sized XRB complements the ceiling-mounted shield and is proportionally the most effective in the projections where the ceiling-mounted shield have least effect. The addition of flaps to the ceiling mounted screen may provide some additional benefit [8].

### Annual operator dose reduction with an XRB

Our data show that adding an optimally placed, rather large (60 cm x 60 cm), 0.5mm lead equivalent XRB to a typical protection setup with a ceiling- and table-mounted shield, could reduce yearly operator dose at shoulder height by 94.9%. This is far better than the 20–76% that have previously been described in clinical studies [4–8]. However, in these studies, blanket position was not standardized, and dislodgement of the XRB or suboptimal positioning of the ceiling-mounted shield may have contributed to lesser shielding effect. Furthermore, some of these studies used single usage sterile XRBs that typically measure only 40 cm x 40 cm and offer 0.125 to 0.25 mm lead equivalent protection. It should be noted that in our measurements, the relatively high doses observed in the standard and no shielding setup likely reflect fixed positioning of the dosimeter to detect the maximum operator dose during a procedure. However, this does not affect the relative benefit of XRB. Optimizing existing XRB design is likely to be a promising path for reducing operator dose with relatively low cost and logistic challenges.

### Perspectives

Use of an optimized XRB can substantially reduce operator dose and is a particularly attractive measure in a field of much concern. It is easily incorporated into existing workflows as it adds minimal procedure time and cost. Compared to more comprehensive shielding solutions it does not need any physical alteration of the cath lab and can be used in a low-resource setting. While we have primarily discussed cardiac procedures, a similar approach could potentially be employed in a variety of medical fields including vascular as well as abdominal and orthopedic surgery.

### Limitations

This article describes an idealized lab setup to assess and improve radiation protection in the cath lab. Further clinical validation should be the subject of future studies. The present study was not designed to assess whether adding an XRB to a shielding setup influences patient dose.

## Conclusion

Adding an XRB of sufficient size can be highly effective at reducing relative operator dose across all angiographic projections and may substantially reduce annual operator dose. An XRB is a low threshold measure that can easily be incorporated into existing workflows. The benefit is largest in the left and cranial projections that are responsible for an estimated 86% of operator dose in our clinical practice. Optimized XRB placement is required in order to prevent radiation from the gap between the patient- and a ceiling- mounted shield.

## Supporting information

S1 Fig Mechanism of action of an X-ray blanket on reducing operator exposure to scatter radiation. Most of the photons of the primary beam are absorbed in the patient. Only a small fraction traverses the patient and creates an X-ray image when it reaches the image detector. The operator is not exposed to the primary beam, but to scatter radiation that occurs when the primary beam interacts with patient tissue (A). Placing an X-ray blanket over the patient shields the operator from scatter radiation (B).
(TIF)

S2 Fig XRB positioning and relative operator dose. To investigate the importance of correctly positioning the ceiling-mounted-shield (CMS) and the X-ray blanket (XRB), four setups were compared in anteroposterior projection to a setup with only table-mounted shield (referred to as "No shielding). In setup A, the CMS was positioned close to the patient and relative operator dose was measured to 35.2% compared to no shielding. With the addition of the XRB positioned 15 cm caudally to the CMS (setup B) relative operator dose was 31.9%, indicating only a small additional shielding effect of the XRB when placed too caudally. With the XRB well-positioned (setup C) close to the image detector and the CMS raised 15cm above de patient relative operator dose was 5.7%. With an optimally placed CMS and XRB (setup D) relative operator dose was 1.5% compared to no shielding.
(TIF)

S3 Fig Descriptive terms of C-arm angulations. C-arm angulation is described by the direction in which the C-arm detector above the patient is tilted. If the X-ray detector is tilted towards the head the projection is termed cranial (CRAN), towards the feet caudal (CAUD), and left or right anterior oblique (LAO/RAO) according to tilt in the left-right direction. Combinations are also possible such as RAO-CRAN.
(TIF)

S1 Table Angiographic projections, C-arm angulation, and percent DAP. (PDF)

S2 Table Estimated annual operator dose according to angiographic projection and shielding setup. (PDF)

S1 File Data file containing C-arm angulation and DAP for each individual exposure and detailed scatter radiation measurement data from the X2 sensor. (ZIP)

## Abbreviations and acronyms

AP: Anteroposterior (projection)
CAUD: Caudal (projection)
cath lab: Cardiac catheterization lab
CRAN: Cranial (projection)
DAP: Dose Area Product
LAO: Left Anterior Oblique (projection)
LAO90: Left Anterior Oblique 90° (projection)
mSv: milliSievert
PCI: Percutaneous Coronary Intervention
RAO: Right Anterior Oblique (projection)
RDSR: Radiation Dose Structured Report
mGy: milliGray
XRB: X-ray Blanket
