# Supplementary figures and images for: Effect of an optimized X-ray blanket design on operator radiation dose in cardiac catheterization based on real-world angiography
Source: PLoS One. 2022 Nov 10;17(11):e0277436. doi: 10.1371/journal.pone.0277436 (PMC9648827; doi:10.1371/journal.pone.0277436)

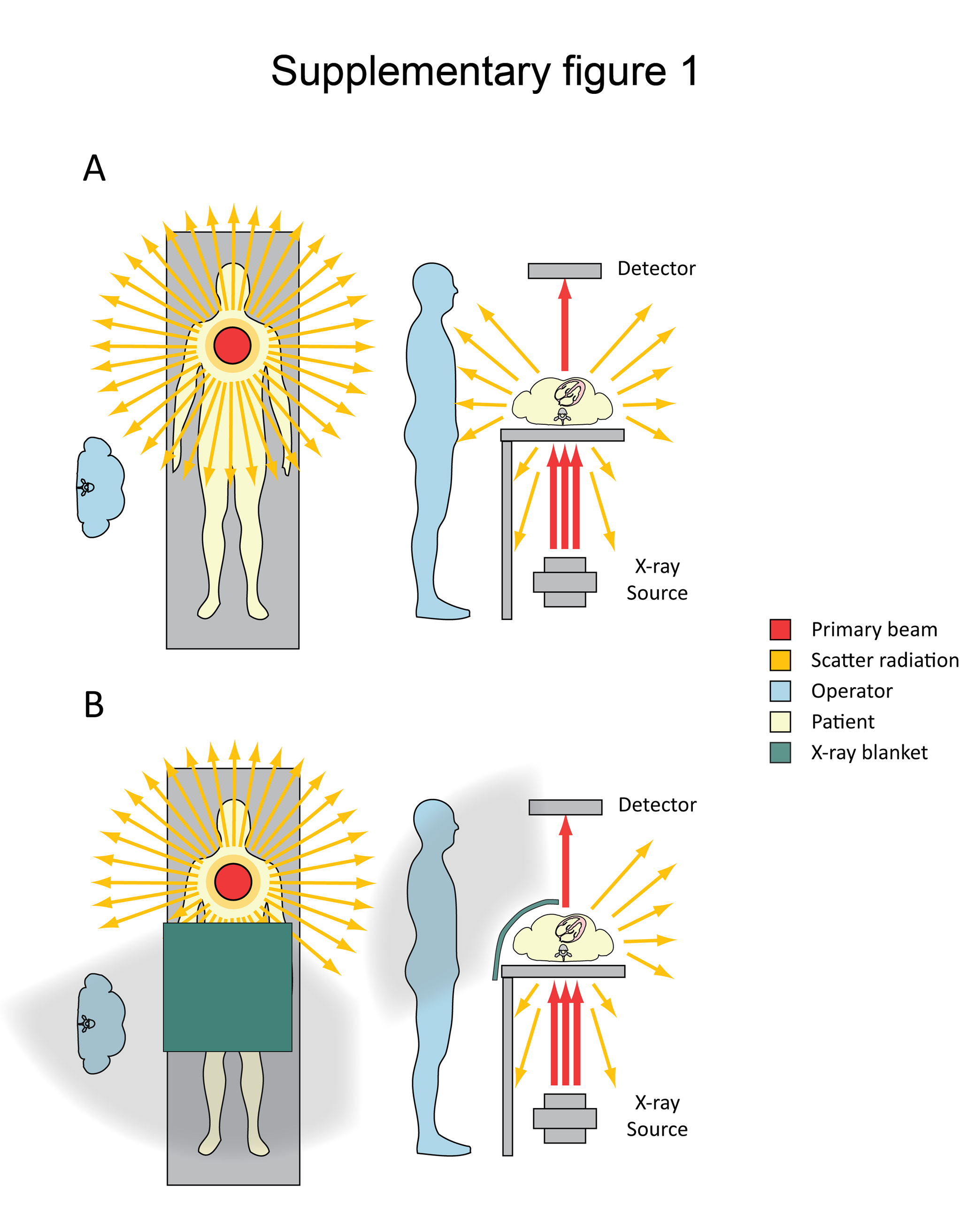

Supplement: S1 Fig — Most of the photons of the primary beam are absorbed in the patient. Only a small fraction traverses the patient and creates an X-ray image when it reaches the image detector. The operator is not exposed to the primary beam, but to scatter radiation that occurs when the primary beam interacts with patient tissue (A). Placing an X-ray blanket over the patient shields the operator from scatter radiation (B). (TIF) [file pone.0277436.s001.tif]

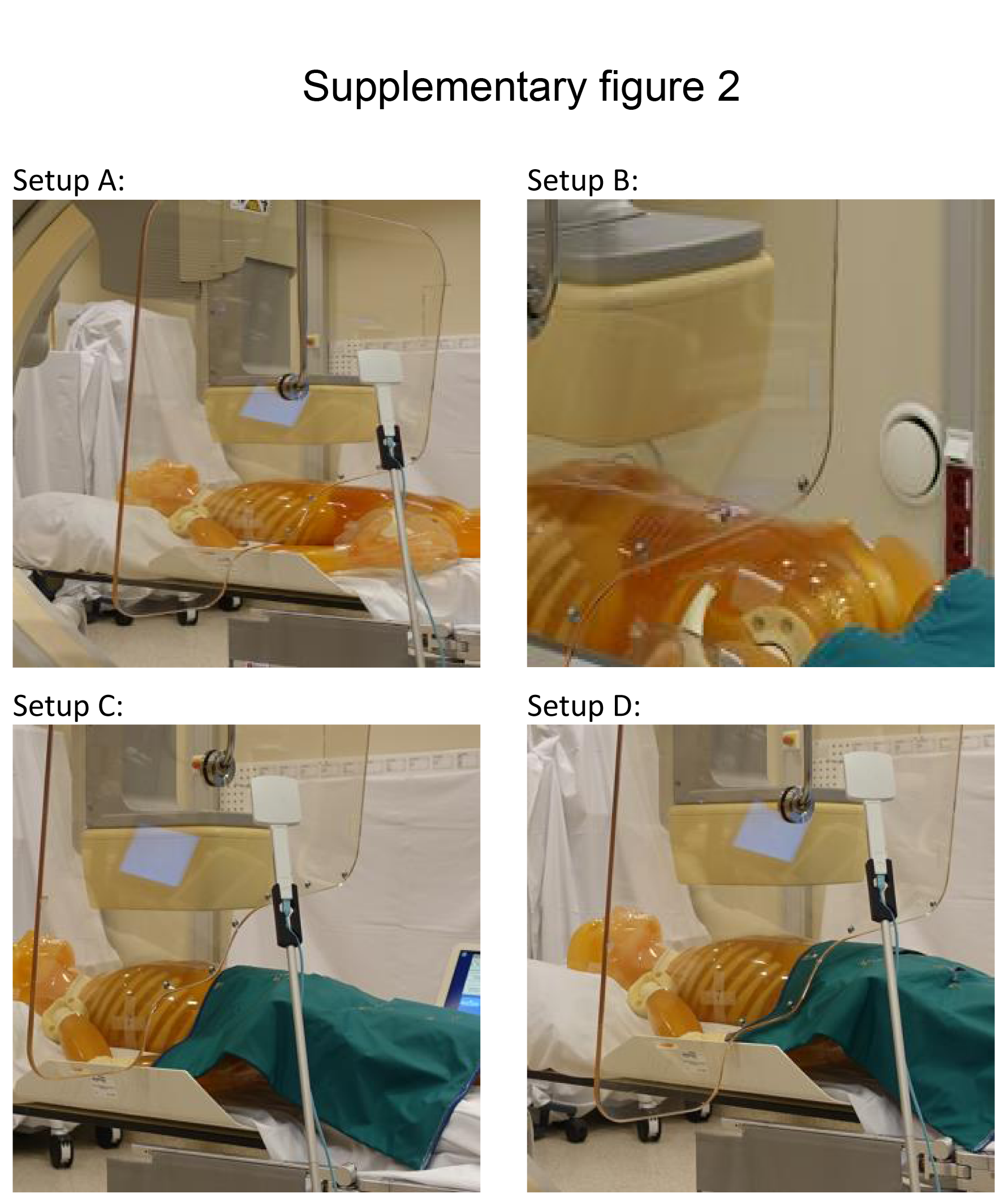

Supplement: S2 Fig — To investigate the importance of correctly positioning the ceiling-mounted-shield (CMS) and the X-ray blanket (XRB), four setups were compared in anteroposterior projection to a setup with only table-mounted shield (referred to as "No shielding). In setup A, the CMS was positioned close to the patient and relative operator dose was measured to 35.2% compared to no shielding. With the addition of the XRB positioned 15 cm caudally to the CMS (setup B) relative operator dose was 31.9%, indicating only a small additional shielding effect of the XRB when placed too caudally. With the XRB well-positioned (setup C) close to the image detector and the CMS raised 15cm above de patient relative operator dose was 5.7%. With an optimally placed CMS and XRB (setup D) relative operator dose was 1.5% compared to no shielding. (TIF) [file pone.0277436.s002.tif]

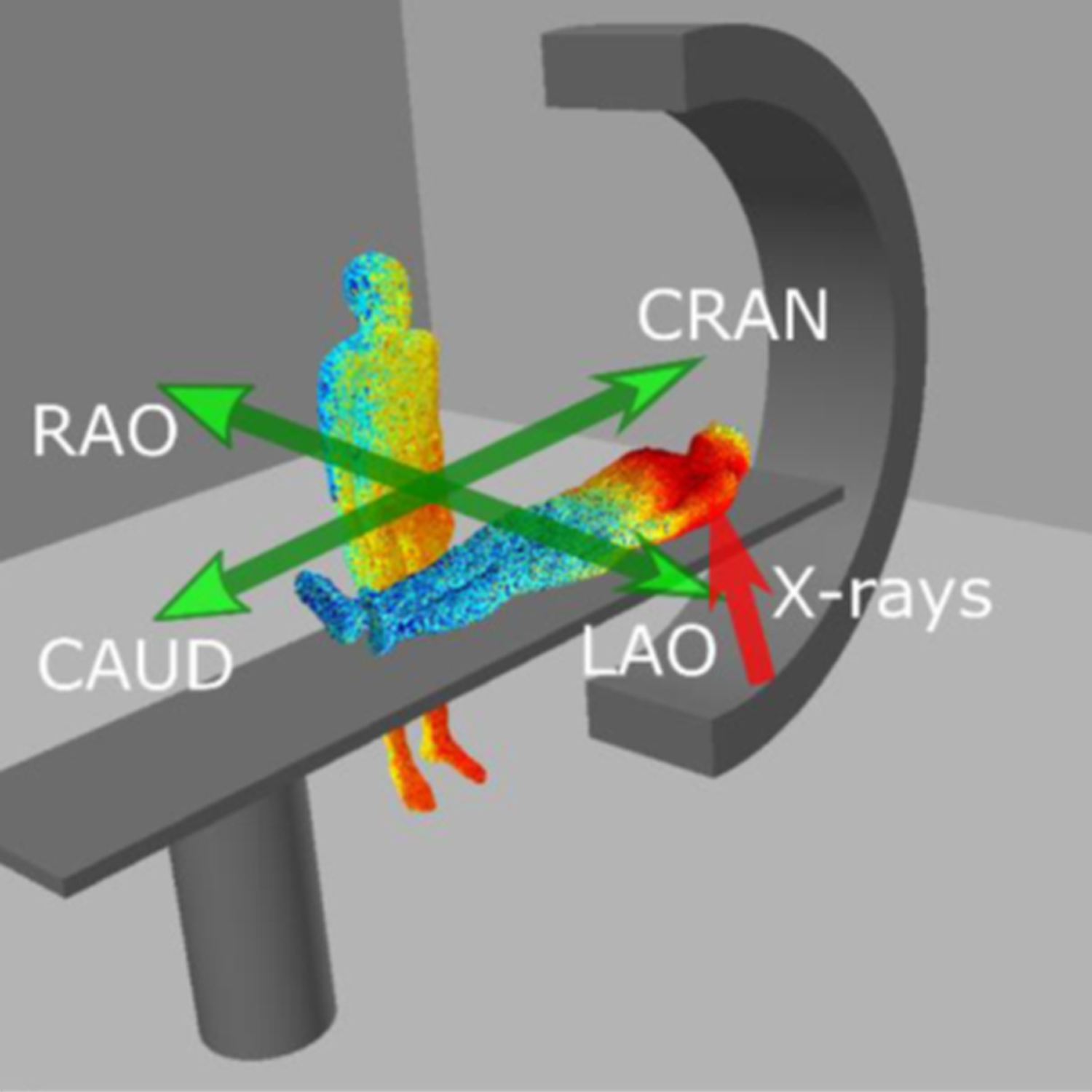

Supplement: S3 Fig — C-arm angulation is described by the direction in which the C-arm detector above the patient is tilted. If the X-ray detector is tilted towards the head the projection is termed cranial (CRAN), towards the feet caudal (CAUD), and left or right anterior oblique (LAO/RAO) according to tilt in the left-right direction. Combinations are also possible such as RAO-CRAN. (TIF) [file pone.0277436.s003.tif]
