# Supplementary material for: Effect of an optimized X-ray blanket design on operator radiation dose in cardiac catheterization based on real-world angiography
Source: PLoS One. 2022 Nov 10;17(11):e0277436. doi: 10.1371/journal.pone.0277436 (PMC9648827; doi:10.1371/journal.pone.0277436)
Supplement: S1 Table — (PDF) [file pone.0277436.s004.pdf]

Supplementary table 1: Angiographic projections, C-arm angulation, and percent DAP.

Projection	n	RAO-LAO	CRAN-CAUD	% DAP
LAO	172343	27	0	21.8
RAO-CRAN	105101	-31	27	14
LAO-CRAN	67549	24	25	11.8
LAO-CAUD	58268	28	-28	11.4
AP	157195	0	0	9.8
CAUD	62441	0	-33	9.4
RAO-CAUD	62555	-28	-24	8.1
CRAN	50510	1	31	7.4
RAO	45524	-29	0	5.8
LAO90	2641	89	-1	0.6

All exposures from 7567 procedures accumulated over three years were grouped into ten angiographic projections. n = number of exposures. RAO-LAO = mean angle in the right-to-left direction. 0º is the center, negative numbers represent right (RAO) tilt, and positive numbers left (LAO) tilt. CRAN-CAUD = mean angle in the cranio-caudal (CRAN-CAUD) direction where negative numbers indicate caudal tilt and positive numbers cranial tilt. % DAP is the percent DAP given to the patient in each projection.
