# Supplementary material for: Effect of an optimized X-ray blanket design on operator radiation dose in cardiac catheterization based on real-world angiography
Source: PLoS One. 2022 Nov 10;17(11):e0277436. doi: 10.1371/journal.pone.0277436 (PMC9648827; doi:10.1371/journal.pone.0277436)
Supplement: S2 Table — (PDF) [file pone.0277436.s005.pdf]

Supplementary table 2: Estimated annual operator dose according to angiographic projection and shielding setup

Projection	% DAP	Annual DAP per operator mGycm²	Projected annual dose per operator (mSv)		
			No shielding	Standard	XRB
AP	9.8	1.7E+06	4.78	0.75	0.07
CAUD	9.4	1.6E+06	6.09	0.14	0.06
CRAN	7.4	1.3E+06	3.68	1.50	0.05
LAO	21.8	3.7E+06	25.36	5.59	0.24
LAO-CAUD	11.4	1.9E+06	11.67	0.37	0.11
LAO-CRAN	11.8	2.0E+06	14.20	5.91	0.10
LAO90	0.6	1.0E+05	2.00	0.01	0.00
RAO	5.8	9.8E+05	2.43	0.15	0.03
RAO-CAUD	8.1	1.4E+06	0.87	0.06	0.02
RAO-CRAN	14	2.4E+06	4.45	0.56	0.08
Sum	100	1.7E+07	75.53	15.03	0.77

Annual DAP per operator is the estimated DAP in $mGycm^2$ based on a yearly case load of 500 cases and mean DAP per procedure 36102 $mGycm^2$. It is distributed to each angiographic projections according to the percentage in which they were used. The projected annual dose per operator is calculated by multiplying the annual DAP per operator with the measured relative operator dose (mSV/DAP) in each projection and shielding setup. DAP = Dose area product, XRB = X-ray blanket.
